# Intercellular communication between extracellular vesicles from conditioned macrophages and breast cancer cells drives endocrine therapy resistance

**DOI:** 10.3389/fcell.2025.1548724

**Published:** 2025-06-03

**Authors:** María C. Rodriguez-Baili, Miguel Palma-Cobo, César G. Prucca, María Yáñez-Mó, German A. Gil

**Affiliations:** ^1^ Departamento de Química Biológica Ranwel Caputto, Facultad de Ciencias Químicas, CONICET, Universidad Nacional de Córdoba-CIQUIBIC, Córdoba, Argentina; ^2^ Centro de Biología Molecular Severo Ochoa (CBMSO), Instituto de Investigación Sanitaria La Princesa (IIS-IP), Instituto Universitario de Biología Molecular (IUBM), Universidad Autónoma de Madrid, Madrid, Spain

**Keywords:** breast cancer, estrogen receptor-positive (ER+), extracellular vesicles (EVs), endocrine resistance, tumor-associated macrophages (TAMs), epithelial-mesenchymal transition (EMT), cancer stem cells (CSS), tumor microenvironment (TME)

## Abstract

**Introduction:**

Breast cancer is a leading cause of cancer-related mortality among women, with nearly 70% of cases being estrogen receptor-positive (ER+). While endocrine therapies, such as tamoxifen, have significantly improved patient outcomes, resistance—whether intrinsic or acquired—remains a major clinical challenge that limits treatment efficacy. Emerging evidence suggests that endocrine resistance is often driven by the presence and expansion of cancer stem cells (CSCs), which contribute to recurrence, metastasis, and therapeutic failure. The tumor microenvironment (TME), including immune cells like macrophages, soluble factors, and extracellular vesicles (EVs), plays a crucial role in promoting tumor progression and therapy resistance. EVs are small lipid bilayer-bound particles that facilitate intercellular communication by transferring bioactive cargo capable of reprogramming recipient cells.

**Methods:**

To investigate the role of macrophage-derived EVs in endocrine resistance, we isolated EVs from TNF-α-conditioned macrophages (TNF EVs) and treated MCF-7 ER+ breast cancer cells with these vesicles. We assessed changes in proliferation, migration, epithelial-mesenchymal transition (EMT), CSC-like properties, and tamoxifen resistance. Additionally, we evaluated whether tumor-derived EVs modulate macrophage polarization by analyzing the expression of PD-1 and other immunomodulatory markers.

**Results:**

TNF EV-treated MCF-7 cells showed significantly increased proliferation, enhanced migratory behavior, and morphological changes associated with EMT. Importantly, treated cells developed a stem-like phenotype, characterized by a larger CD44^High^/CD24^Low^ subpopulation and improved spheroid-forming ability. These features correlated with sustained proliferation even in the presence of tamoxifen, supporting the development of endocrine resistance. Furthermore, EVs derived from tumor cells triggered macrophage polarization toward a tumor-associated macrophage (TAM) profile, with increased PD-1 expression, indicating a role in immune suppression and tumor immune evasion.

**Discussion:**

These findings emphasize the dual role of TNF-α-conditioned macrophage-derived EVs in driving both endocrine resistance and immune modulation in ER+ breast cancer. By promoting stemness, EMT, and tamoxifen resistance, as well as inducing immunosuppressive macrophage polarization, these EVs emerge as key contributors to tumor progression. Our study highlights the therapeutic potential of targeting EV-mediated communication to overcome endocrine resistance and enhance clinical outcomes for ER+ breast cancer patients. This work establishes a critical framework for future studies aimed at harnessing EVs as therapeutic targets or biomarkers in breast cancer management.

## Graphical Abstract

**Figure:**
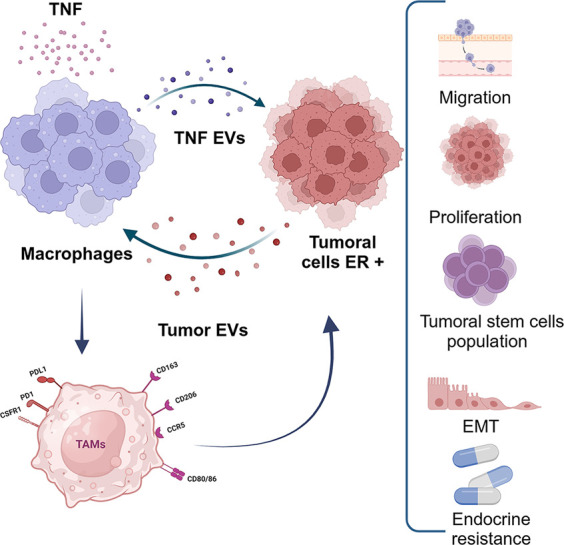
TNF-α-conditioned macrophages secrete EVs that MCF-7 cells internalize, driving increased proliferation, migration, cancer stem cell expansion, epithelial-mesenchymal transition (EMT), and endocrine resistance. Additionally, a feedback loop emerges: ER+ tumor cells release EVs that polarize macrophages towards a tumor-associated macrophage (TAM) phenotype, reinforcing a pro-tumoral microenvironment and accelerating disease progression.

## 1 Introduction

Breast cancer remains one of the most prevalent malignancies worldwide and a leading cause of cancer related mortality among women. More than 70% of diagnosed breast tumors are estrogen receptor-positive (ER+) (DeSantis et al., 2019). These tumors predominantly depend on the activation of estrogen receptors ERα and ERβ by the steroid hormone estradiol (E2), which drives the proliferation of both normal and neoplastic breast epithelial cells (Hanker et al., 2020). Upon binding E2, the estrogen receptor forms a homodimer, translocate to the nucleus, and binds to estrogen response elements (EREs) in the DNA, triggering the transcription of proliferative genes such as MYC and Cyclin D1 (Prall et al., 1998; Clusan et al., 2023). Beyond direct DNA binding, estrogen receptors can also be activated indirectly through crosstalk with other transcription factors, further modulating gene expression. Ligand independent activation of ER through phosphorylation by kinases downstream of growth factor signaling pathways has also been documented (Weigel and Zhang, 1998).

ER signaling is a critical target in breast cancer therapy, with selective estrogen receptor modulators (SERMs), selective estrogen receptor degraders (SERDs), and aromatase inhibitors (AIs) serving as the foundation for adjuvant treatment for ER+ breast cancer. However, a significant challenge in clinical management is the development of endocrine resistance, either *de novo* or acquired during treatment, leading to treatment failure in a subset of patients (Johnston, 2010). Importantly, understanding the role of the tumor microenvironment (TME) in underlying resistance mechanisms is key to improving therapeutic outcomes.

The TME plays a central role in cancer progression and resistance mechanisms. It consists of a dynamic milieu of extracellular matrix (ECM), soluble factors, stromal cells, and immune cells, including macrophages, lymphocytes, and fibroblasts (Bhowmick et al., 2004; Kim et al., 2005). Among immune cells, tumor-associated macrophages (TAMs) represent 30%–50% of immune infiltrates in breast cancer (Hu et al., 2021; Li et al., 2023) and are associated with poor prognosis across multiple tumors, including breast, cervical, bladder, and glioma (Bingle et al., 2002; Coussens et al., 2013; Fortis et al., 2017; Honkanen et al., 2019; Komohara et al., 2016; Locati et al., 2020; Moeini and Niedźwiedzka-Rystwej, 2021). TAMs exhibit remarkable plasticity, adapting to signals from the TME, macrophages can polarize into either pro-inflammatory (M1) or anti-inflammatory (M2) phenotypes (Braun et al., 2021; Etzerodt et al., 2020; Movahedi et al., 2010). This plasticity underscores their dual role in both suppression and promotion of tumor growth.

An important mediator of TAMs function is Tumor Necrosis Factor-alpha (TNF-α), a pro-inflammatory cytokine that promotes both tumor growth and inflammation in the breast cancer microenvironment (Cruceriu et al., 2020). Secreted mainly by TAMs and tumor cells, TNF-α plays a significant role in breast cancer progression by facilitating epithelial-to-mesenchymal transition (EMT), metastasis, and endocrine resistance (Egberts et al., 2008; Sethi et al., 2008; Stathopoulos et al., 2007; Zins et al., 2007). Its effects are mediated through the activation of the NF-κB and ERK signaling pathways, highlighting its role as a “master regulator” of inflammation and macrophage activation (Parameswaran and Patial, 2010). Our earlier research showed that TNF-α conditioned macrophages and their secreted factors boost the proliferation, migration, invasion and endocrine resistance of ER+ breast cancer cells (Castellaro et al., 2019).

A new mechanism of tumor-stroma communication involving extracellular vesicles (EVs) has emerged. These small membrane-enclosed particles, secreted by almost all cell types, were once considered waste disposal systems (Couch et al., 2021; Kosaka et al., 2013; Raposo et al., 1996). EVs are classified into *exosomes*, *microvesicles*, and *apoptotic bodies* according to their size and origin. Exosomes, which range from 30 to 150 nm, originate when multivesicular bodies (MVBs) fuse with the plasma membrane, while microvesicles bud directly from the membrane (Buzas, 2023; Colombo et al., 2014; Tkach et al., 2017; Yáñez-Mó et al., 2015). Although the molecular machinery that regulates EV biogenesis is not fully understood, members of the Rab family of GTPases play a crucial role in vesicle trafficking and secretion, with Rab27a/b being essential for exosome release (Ostrowski et al., 2010).

The biological importance of EVs lies in their capacity to transfer a variety of proteins, lipids, and nucleic acids to target cells, which can trigger significant changes in their phenotype. The cargo within EVs depends on the source of the cells and their physiological or pathological conditions, highlighting EVs as potential biomarkers for liquid biopsies (Minciacchi et al., 2015). Additionally, EVs remain notably stable in biological fluids such as plasma, saliva, and urine, making them appealing for noninvasive diagnostic applications (Colombo et al., 2014; Caby et al., 2005; Kosaka et al., 2010; Palanisamy et al., 2010; Pisitkun et al., 2004; Poliakov et al., 2009; Torregrosa Paredes et al., 2012). The selection of exported proteins in EVs is influenced by the state of the cell of origin and the subcellular compartment from which they originate (Corrado et al., 2013; Muralidharan-Chari et al., 2009). The proteins frequently used as vesicle markers or identifiers are also involved in their biogenesis, including Alix, Tgs 101, ceramide, flotillin, Rab, and tetraspanin family members (Colombo et al., 2013; Romancino et al., 2013; Trajkovic et al., 2008). Notably, tetraspanins CD9, CD81, and CD63 participate in endosomal vesicle trafficking (Abache et al., 2007; Pols and Klumperman, 2009; Toribio and Yáñez-Mó, 2022).

Given the emerging significance of EVs in mediating intercellular communication, their role in cancer progression has attracted considerable attention. Tumor-derived EVs have been shown to transport oncogenic cargo to stromal and immune cells, facilitating critical processes such as tumor proliferation, invasion, metastasis, and therapeutic resistance (Bebelman et al., 2018; Maacha et al., 2019; Rak and Guha, 2012). This study focused on the impact of EVs secreted by TNF-α conditioned macrophages (TNF EVs) on ER+ breast cancer cells, specifically the MCF-7 cell line. Our results revealed that TNF EVs significantly enhanced the proliferation, migratory capacity, and EMT of MCF-7 cells and induced a cancer stem cell-like phenotype. Importantly, TNF EVs also conferred resistance to Tamoxifen, a selective estrogen receptor modulator, in these cells, highlighting their role in endocrine therapy resistance.

Additionally, we explored the mutual relationship between breast cancer cells and the TME by assessing how MCF-7-derived EVs influence macrophage polarization. Our findings revealed that EVs from MCF-7 cells (MCF-7 EVs) encouraged a pro-tumorigenic M2 macrophage phenotype marked by increased PD-1 expression, contributing to an immunosuppressive and tumor-supporting environment. These findings spotlight the pivotal importance of EV-mediated communication in promoting therapeutic resistance, indicating that disrupting EV signaling may offer a groundbreaking new approach for treating ER+ breast cancer.

## 2 Materials and methods

### 2.1 Cell culture

Human breast cancer cells MCF-7 and monocytes THP-1 were obtained from the American Type Culture Collection (ATCC). MCF-7 cells were maintained in Dulbecco’s Modified Eagle Medium (DMEM) (Thermo Fisher), supplemented with 10% Fetal Bovine Serum (FBS) (Gibco), 1X non-essential amino acids MEM (Gibco), and penicillin-streptomycin-neomycin mix as an antibiotic (PSN) (Gibco) at 37°C in a 5% CO_2_ controlled atmosphere. The monocytic THP-1 cell line was maintained in RPMI 1640 medium (Gibco), supplemented with 10% FBS and 1X PSN as an antibiotic mix. Control for *Mycoplasma* contamination was performed periodically using a PCR-based method with internal loading control. Cell lines were used for experimentation for no more than 20 passages from the main frozen stock.

### 2.2 Macrophage differentiation and conditioning

Differentiation of the THP-1 monocytic cell line was induced according to an adaptation of the protocol from Genin (Genin et al., 2015). Briefly, THP-1 cells were cultured with 10 ng/mL of phorbol-12-myristate-13-acetate (PMA) for 48 h in RPMI 1640 medium with the addition of 10% FBS and 1X PSN. After differentiation, macrophages were treated with 1 ng/mL of human recombinant TNF-α for 6 h at 37°C in a 5% CO_2_ controlled atmosphere to obtain conditioned macrophages.

### 2.3 Macrophages polarization

The THP-1 monocytic cell line was differentiated into macrophages M0 with 10 ng/mL of PMA in RPMI 1640 medium containing 10% FBS and 1X PSN for 48 h. They were then polarized to M1 using 20 ng/mL of Interferon-gamma (IFN-γ) (eENZYME LCC) and 10 pg/mL of LPS (Sigma-Aldrich) for 48 h in RPMI 1640 medium with 10% FBS and 1X PSN. To obtain M2 macrophages, cells were incubated with 80 ng/mL of IL-4 (Sigma-Aldrich) for 48 h in RPMI 1640 medium supplemented with 10% FBS and 1X PSN.

### 2.4 EVs isolation by ultracentrifugation

Cells of interest were cultured in a complete medium until they reached 80% confluence. Subsequently, the culture medium was replaced with a serum-free medium to minimize contamination from serum-derived vesicles. After 24 h of incubation, the culture medium was collected and centrifuged at 300 × g for 10 min to remove cellular debris. This process was repeated twice. The resulting supernatant was centrifuged at 2000 × g for 20 min to isolate the 2 K subpopulation. Subsequently, the supernatant was ultracentrifuged at 10,000 × g for 40 min to obtain the 10 K subpopulation containing apoptotic bodies and microvesicles. The remaining supernatant was ultracentrifuged at 100,000 × g for 60 min to isolate the 100 K subpopulation, enriched with small EVs such as exosomes. A final ultracentrifugation step at 200,000 × g for 60 min was performed to obtain the 200 K subpopulation. At each step, EVs were washed with PBS at the same speed and time corresponding to each subpopulation. All pellets were resuspended at a final concentration of one million secreting cells per μL of PBS. Before resuspension, the secreting cells were quantified to determine their total number. The different pellets were stored and aliquoted into 20–30 μL fractions to prevent repeated freeze-thaw cycles and maintain vesicle integrity. All ultracentrifugation procedures were performed using a Beckman Coulter ultracentrifuge with a 70.1 Ti rotor (K factor = 42).

Fraction 2 K was omitted for the EV pool isolation, and the remaining supernatant was directly subjected to ultracentrifugation at 100,000 × g for 60 min. The resulting pellet was washed and resuspended at 1 million secreting cells per μL of PBS, designated as EVs.

### 2.5 Size exclusion chromatography (SEC)

Cells of interest were cultured in EV-depleted FBS (previously ultracentrifuged at 100,000 g overnight) for 5 days or 24 h for macrophages. The culture medium was centrifuged at 300 x g for 15 min, after which the supernatant was collected and centrifuged again at 3,000 × g for 30 min. The resulting supernatant was loaded into a 100 kDa pore size Amicon filter (Merck) and concentrated by centrifugation at 2,000 × g for 20 min until a total volume of 500 μL was obtained. The concentrated medium was then loaded into the top of a Sepharose 2BCL column (A-1021S-500 ABT BEADS), and the sample was eluted with filtered 1X PBS, collecting 25 fractions of 500 μL each. The fractions were subjected to Dot Blot analysis using a specific extracellular vesicle marker to select those corresponding to vesicles and soluble protein fractions.

### 2.6 Dot blot of SEC fractions

From the 25 fractions collected after SEC, 1 μL of each was placed onto a nitrocellulose membrane. The membrane was blocked with 5% w/v milk in PBS containing 0.05% v/v Tween 20 (PBST) for 10 min, followed by incubation with an antibody against tetraspanin (hybridoma supernatant, undiluted) CD63 (Tea3.10) (Yáñez-Mó et al., 1998) for 40 min at room temperature (RT). After washing the membrane, it was incubated with a secondary antibody for detection using an infrared detector, such as Odyssey. This process allowed for the identification of fractions containing EVs.

### 2.7 Nanoparticle tracking analysis (NTA)

The size distribution and concentration of isolated EVs were assessed by measuring the Brownian motion rate using a NanoSight LM10 system (Malvern) equipped with a 405 nm laser. For each sample, at least two 30-s videos were recorded and analyzed, with a detection threshold value equal to 10. Data was obtained using a shutter speed 345 and a camera gain 1.00. Measurements were performed at RT. The recorded videos were analyzed using NTA software version 2.2. Samples were diluted 1/100 in filtered PBS to ensure the particle concentration remained within the dynamic range of the NTA software.

### 2.8 EV uptake assay

EVs isolated from TNF-α conditioned macrophages were isolated using size exclusion chromatography (SEC) and quantified using nanoparticle tracking analysis (NTA). For fluorescence labeling, the EVs were incubated overnight at 4°C with Alexa Fluor 633 C5-Maleimide at a final concentration of 25 μM (Toribio and Yáñez-Mó, 2023). An additional SEC purification step was performed to eliminate unbound dye. The purified labeled EVs were then analyzed by Dot Blot and NTA to verify their concentration and labeling efficiency. One day before the uptake assay, recipient cells were seeded at 150,000 cells per well in a 24-well plate with 500 μL of medium containing EV-depleted serum. Labeled EVs were added at 16,000 vesicles per cell and incubated with the cells for 2 h at 37°C and 5% CO_2_ in the dark. After incubation, the cells were harvested using trypsin and examined for EV uptake via flow cytometry (BD Canto II cytometer). Cells not receiving labeled EVs served as negative controls, with fluorescence detection set on the 633 nm channel.

### 2.9 Cell lines Western blot

Western blot assays were performed to assess changes in the expression levels of different proteins. Samples were previously lysated with Triton X-100 and protease inhibitors to a final concentration 1X. Lysates were collected in adequately labeled 1.5 mL tubes and centrifuged at 15,000 rpm for 15 min. The supernatants were transferred to newly labeled 1.5 mL tubes and kept at −20°C until further processing. 6X sample buffer (60 mM TRIS-HCl, pH 6.8, 25% v/v glycerol, 2% w/v SDS, and 0.1% w/v bromophenol blue) and 1 M Dithiothreitol (DTT) were added in appropriate amounts for a final concentration of 1X in the cell lysates. Samples were boiled for 5 min at 100°C. Electrophoresis was carried out with 30 μg of protein per lane seeded in 10% w/v acrylamide/bisacrylamide SDS-PAGE in running buffer (25 mM TRIS, 192 mM Glycine, 0.1% SDS), pH 8.3 at 100 V, under denaturing (SDS) and reducing (DTT) conditions. Proteins were transferred to nitrocellulose membranes (Santa Cruz Biotechnology, 0.22 μm pore size) in Tris buffer (25 mM TRIS, 192 mM Glycine, 20% v/v ethanol), pH 8.3 at 300 milliamps for 1 h. Subsequently, the membranes were blocked with 5% w/v milk in PBS containing 0.05% v/v Tween-20 (PBST) for 1 h at RT and incubated with the primary antibody overnight at 4°C. The following primary antibodies were used at a dilution of 1/500: anti-E-cadherin (BD Biosciences), anti-Tubulin (Sigma-Aldrich), and anti-Beta-catenin (Sigma-Aldrich). The secondary antibodies were IRDye 800 CW mouse or IRDye 700 CW rabbit (1/10,000) (LI-COR). Both dilutions of primary and secondary antibodies were made in PBST in 5% w/v, not fat milk. Membranes were visualized and quantified using the Odyssey infrared imaging system (LI-COR).

### 2.10 EVs Western blot

Western blot assays were performed to assess the presence or absence of specific proteins used to identify extracellular vesicles, thus verifying proper isolation and characterizing the population of vesicles derived from cell lines. As tetraspanins are glycosylated, Western blot was performed as detailed for cell lines but under denaturing (SDS) and non-reducing conditions (without DTT) using 4X sample buffer (0.125 M Tris-HCl pH 6.8, 8% w/v SDS, 40% v/v glycerol, and 0.02% bromophenol blue). The following primary antibodies were used at a dilution of 1/500: anti-CD63 (Invitrogen), anti-CD81 (Invitrogen), anti-CD9 (Thermo Fisher), and anti-gp96 (Novus Biologicals). The secondary antibodies were IRDye 800 CW mouse or IRDye 700 CW rabbit (1/10,000) (LI-COR). Both dilutions of primary and secondary antibodies were made in 5% w/v not fat milk in PBST. Membranes were visualized and quantified using the Odyssey infrared imaging system (LI-COR).

### 2.11 Proliferation assays

7,000 MCF-7 cells per well in 100 μL of the corresponding complete medium were cultured in 96-well plates. The cells were allowed to settle for 24 h, followed by a wash with 1X PBS before incubating them under different experimental conditions: DMEM culture medium (Control) and different concentrations of vesicles (derived from one million and two million initial secreting cells) from macrophages conditioned with TNF-ɑ (TNF EVs). After 24 h, Alamar Blue fluorometric assay (Invitrogen) was performed. The Alamar Blue cell viability reagent is a resazurin-based solution used as an indicator of cellular metabolism by utilizing the reducing capacity of living cells to measure their viability quantitatively. Resazurin is a non-toxic, cell-permeable compound, initially blue and virtually non-fluorescent. Once inside living cells, it is converted to resorufin, a red-colored and highly fluorescent compound. Changes in cell viability can be easily detected using a plate reader that measures absorbance or fluorescence (Rampersad, 2012). The cells were washed twice with 1X PBS; the corresponding medium with 10% v/v Alamar Blue was added and then incubated, protected from light, at 37°C in a 5% CO_2_ controlled atmosphere for at least 4 h. Fluorescence was measured using a Biotek fluorometer at 24 and 48 h post addition of EVs. Measurements were taken at 530/590 nm. The results were expressed as a percentage of proliferation relative to the control.

### 2.12 Wound healing assay

A wound healing assay evaluated the migration of MCF-7 cells in the presence of TNF EVs. For this purpose, 250,000 MCF-7 cells were seeded in a 24-well plate with 500 μL of 1% FBS DMEM and 1X PSN to prevent their proliferation. After 24 h, a wound was created vertically across the monolayer using a tip. The monolayer was then washed with 1X PBS three times to remove detached cells during wound creation, and the medium containing the corresponding vesicle subpopulations was added. The wound was measured 24 and 48 h after the addition of TNF EVs. Cells without vesicle treatment served as a control. Images were captured using a Leica DMI confocal microscope (Leica DMI 8 microscope, with Leica monochromatic camera, motorized stage, and Las-X software) utilizing the “Mark and Find” function, which captures images at the same points at different times. The images were processed using ImageJ software and a wound healing assay plugin. The results were expressed as the percentage of wound area relative to the control.

### 2.13 Protrusion quantification

Following the wound healing assay, cells were fixed with 4% paraformaldehyde (PFA) for 10 min, washed three times with 1X PBS, and then stained with Alexa Fluor 568 Phalloidin (Molecular Probes) to visualize actin morphology. After three additional washes with 1X PBS, cells were stained with 1X DAPI to label nuclei for 20 min. Images were captured at 40X magnification using the Leica DMI 8 confocal microscope and processed using ImageJ software.

### 2.14 Tumor stem cell flow cytometry

150,000 MCF-7 cells were cultured in a 24-well plate with 500 µL of complete medium. After 24 h, different treatments were performed: complete DMEM for the control, TNF EVs, and the SF from TNF-conditioned macrophages. Then, they were incubated for an additional 48 h. Cells were trypsinized and then incubated with the corresponding antibodies for 40 min at 4°C. Cells were analyzed using a BD Canto II flow cytometer, with a fluorescence threshold established through controls of unmarked cells and cells individually marked with each antibody. Anti-CD44-APC (Bioscience) and anti-CD24-PECy7 (BD Bioscience) antibodies were used at a ratio of 5 µL per million cells, as indicated in their respective catalogs. Results were analyzed using FlowJo software and expressed as a percentage of the population relative to the control.

### 2.15 Tumor spheroid formation

10,000 MCF-7 cells were seeded in 96-well ultra-low attachment (ULA) round-bottom plates (Corning^®^) in 100 μL of serum-free DMEM supplemented with 10 ng/mL basic fibroblast growth factor (bFGF) (Gibco), 20 ng/mL epidermal growth factor (EGF) (Sigma-Aldrich), and 1X PSN antibiotic mixture. TNF EVs were added simultaneously with the cells. Cultures were maintained for 6 days at 37°C and 5% CO_2_, with half of the medium gently replaced every 3 days to avoid disturbing spheroid integrity (Chen et al., 2022). Spheroid formation was monitored using bright-field microscopy with the Cytiva InCell Analyzer 2500HS system and processed using ImageJ software. Images were acquired at 4X magnification from three independent wells per experimental condition. After 6 days, spheroids were harvested, enzymatically dissociated into single-cell suspensions, and processed for flow cytometric analysis as described in section 2.14.

### 2.16 Endocrine resistance assays

7,000 MCF-7 cells per well were cultured in 100 µL of the corresponding cell line medium on 96-well plates. After 24 h, the cells were washed with 1X PBS and then incubated under different experimental conditions: DMEM without phenol red supplemented with 1% FBS (Control), vesicles from 3 million TNF-conditioned macrophages, and various concentrations of 4-Hydroxytamoxifen (Sigma-Aldrich) (1, 5, 10, 25, 50 µM) (Shagufta, 2018; Abdallah et al., 2020; Seeger et al., 2003). For this assay, DMEM without phenol red (Gibco), 1X PSN, and 1% FBS were added to prevent estrogen receptor stimulation. After 24 h, the Alamar Blue fluorometric assay was performed as described above. Fluorescence was measured using a Biotek fluorometer 24 h after adding EVs and Tamoxifen, and the results were expressed as a percentage of proliferation relative to the control.

### 2.17 Macrophage differentiation assay and PD-1 expression by flow cytometry

150,000 THP-1 cells were differentiated to macrophages M0 using 10 ng/mL PMA for 48 h in a 24-well plate with 500 µL of complete medium. After 48 h, the cells were incubated under different experimental conditions: with a complete medium (Control) or EVs derived from 50 million MCF-7 cells (MCF-7 EVs). After another 48 h of incubation, the cells were harvested using Accutase (Gibco) and stained with anti-PD-1 PECy7 (Biolegend), anti-CD86 APC (Biolegend) to identify M1 macrophages, and anti-CD206 APCCy7 (Biolegend) to identify M2 macrophages. According to the manufacturer’s instructions, flow cytometry antibodies were used at a concentration of 5 µL per million cells. The cells were analyzed by flow cytometry on a Beckton Dickinson BD LSR Fortessa. Cells not incubated with EVs and macrophages polarized to M1 and M2 were used as controls. The results were analyzed using FlowJo software and expressed as a percentage of the population relative to the control.

### 2.18 Antibodies and reagents

We used the following primary antibodies: anti-Tubulin #T9026 (Sigma-Aldrich), anti-E-Cadherin #610181 (BD Biosciences), anti-Beta-catenin #SAB5701390 (Sigma-Aldrich), anti-CD63 #10628D (Invitrogen), anti-CD81 #MA513548 (Invitrogen), anti-CD9 #MA5-31980 (Thermo Fisher), anti-gp96 #NB300-619 (Novus Biologicals), anti-CD44 APC #17-0441-83 (Bioscience), anti-CD24 PECy7 #561646 (BD Bioscience), anti PD-1 PE Cy7 #329918 (Biolegends), anti-CD86 APC #605412 (Biolegends), anti-CD206 APC Cy7 #321120 (Biolegends). The secondary antibodies used were IRDye 800 CW mouse #C70301-02 (LI-COR) and IRDye 700 rabbit #C61110-06 (LI-COR). The reagents used were: Alexa Fluor 568 Phalloidin #A12380 (Molecular Probes), PMA #98139-1MG (Sigma-Aldrich), 4-Hydroxytamoxifen #H7904-5MG (Sigma-Aldrich), Alamar Blue #DAL1025 (Invitrogen), TNF #GF314 (Sigma-Aldrich), IL-4 #GF337 (Sigma-Aldrich), LPS #L6529-1MG (Sigma-Aldrich), IFN-gamma #INF-G2-020P (eENZYME LCC), Alexa Fluor 633 C5-Maleimide #A20342 (Thermo Fisher), FGFb #AA10155 (Gibco), EGF #E4127 (Sigma- Aldrich).

### 2.19 Statistical analysis

The data analysis, assessment of the statistical significance of observed differences, and the presented graphs were generated using GraphPad Prism 8 Software (San Diego, CA, United States). Statistical analysis of differences between the two groups was performed using Student's t-tests. One- or two-way analysis of variance (ANOVA), followed by multiple comparison tests such as Kruskal–Wallis, Sidak, or Dunnett’s test, was employed to determine statistical differences among more than two different groups. Mean ± standard error of the mean (SEM) or standard deviation (SD) (depicted as T) were plotted in parametric analyses. The statistical significance of mean differences is indicated in the figures as: **** (p < 0.0001), *** (p < 0.001), ** (p < 0.01), * (p < 0.05).

## 3 Results

### 3.1 Isolation and characterization of EVs

**FIGURE 1:**
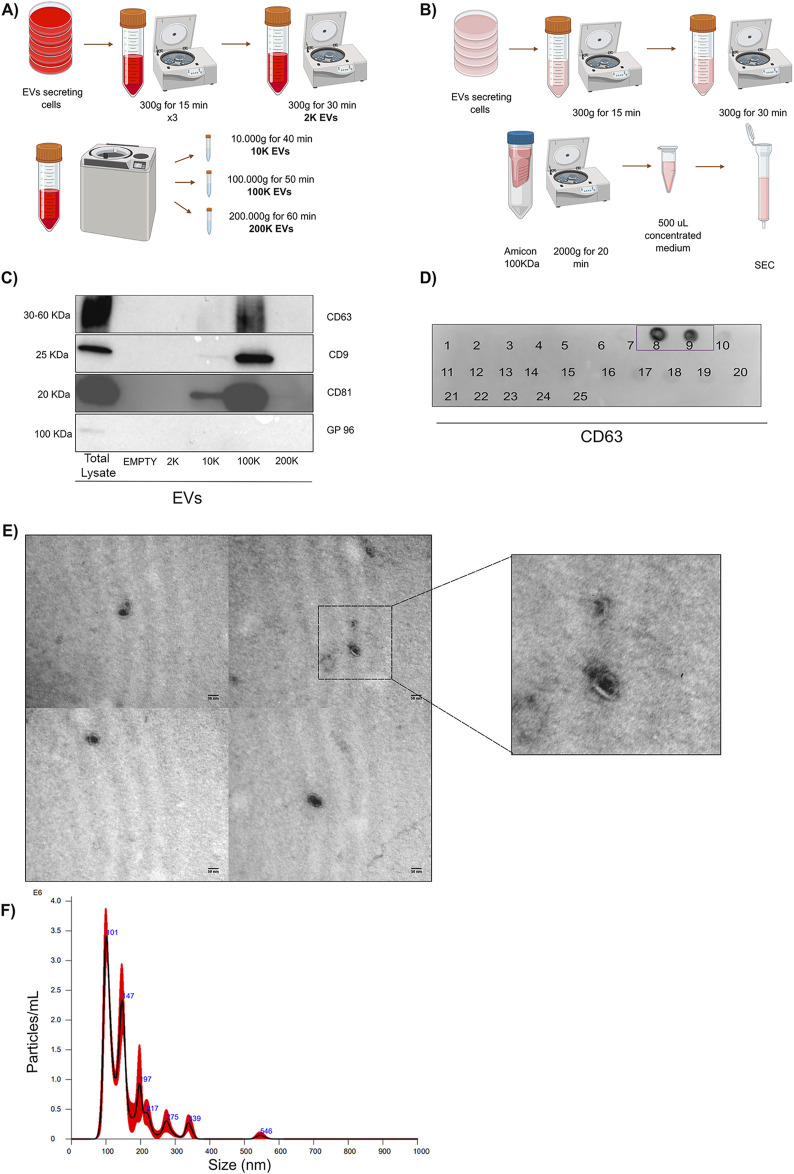
Isolation and characterization of EVs. **(A)** Schematic representation of the ultracentrifugation protocol employed to isolate distinct EVs subpopulations. The pellets collected at 2K, 10K, 100K, and 200 K were sequentially washed in PBS at the respective centrifugation speeds and resuspended to a concentration of 1 million cells per µL. Image generated using BioRender.com. **(B)** Diagram of the SEC method used for EVs isolation. A total of 25, 0.5 mL fractions were collected for further analysis. Image generated using BioRender.com. **(C)** Western blot analysis showing the expression of common EVs surface markers—CD63, CD9, and CD81—confirms EVs’ presence. The absence of gp96 in the EVs lanes indicates minimal contamination from other cellular components. The total cellular homogenate was used as a control. **(D)** Dot blot analysis of the 25 fractions obtained from SEC, probing for CD63 to identify fractions containing EVs. Fractions containing EVs are boxed and numbered accordingly. **(E)** EVs morphology analysis by TEM. EVs display a typical cup-shaped appearance, consistent with the preparation process and negative staining. The scale bar is 50 nm. **(F)** NTA results depict the concentration and size distribution of EVs recovered by SEC, with a particle size range consistent with extracellular vesicles.

In this study, we first focused on isolating and characterizing EVs derived from both TNF-α conditioned macrophages (TNF EVs) and MCF-7 breast cancer cells (MCF-7 EVs). To achieve this, we employed a combination of differential ultracentrifugation and size exclusion chromatography (SEC), two well-established methods for EV isolation (Cocozza et al., 2020). Ultracentrifugation enabled us to isolate distinct EVs subpopulations based on size and density, specifically yielding 2 K, 10 K, 100 K, and 200 K fractions. SEC further refined the separation, allowing us to collect EVs enriched fractions with minimal contamination from other components, such as protein aggregates and non-vesicular structures. The workflow for EV isolation is illustrated (Figures 1A,B). To confirm the purity of our EVs preparations, we conducted Western blotting on the different subpopulations of EVs obtained from the same number of producing cells (20 × 10^6^). Although no specific marker for EVs has been described, the use of tetraspanins CD63, CD81, and CD9 as identifying proteins for EVs is widely accepted (Welsh et al., 2024). Our results demonstrated that these tetraspanins were highly enriched in the smaller EVs fractions, while gp96, a marker for the endoplasmic reticulum, was notably absent. These observations indicate minimal contamination from intracellular compartments, indicating high purity EVs isolation (Figure 1C). In addition, dot blot analysis using anti-CD63 antibodies further validated the presence of EVs in the SEC-obtained fractions (Figure 1D). To visualize the isolated EVs and ensure that their morphology and size were consistent with expectations, we performed transmission electron microscopy (TEM). TEM images confirmed the presence of EVs with the characteristic cup-shaped morphology and an average diameter of 50–150 nm, consistent with small EVs’ size range. (Figure 1E). These results were further corroborated by nanoparticle tracking analysis (NTA), which quantified particles’ size distribution and concentration. NTA results showed a particle size distribution consistent with the TEM observations, and we estimated that approximately 16,000 EVs were added per recipient cell in functional assays described below, ensuring adequate exposure for cellular interaction studies (Figure 1F).

### 3.2 TNF EVs promote proliferation in breast cancer cells

**FIGURE 2:**
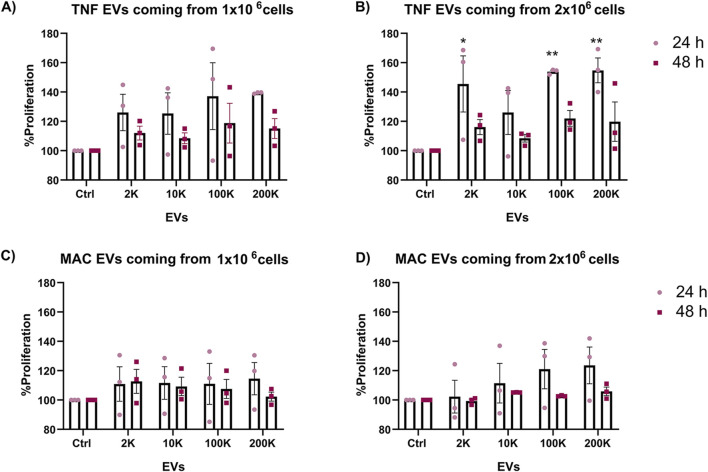
Enhanced proliferation of MCF-7 cells in the presence of TNF EVs. **(A,B)** Proliferation of MCF-7 cells was assessed after 24 and 48 h of treatment with different subpopulations and concentrations of EVs isolated from TNF-α conditioned THP-1 macrophages (TNF EVs). Data from **(A)** TNF EVs derived from one million cells **(B)** two million cells is shown. **(C,D)** Proliferation of MCF-7 cells treated with EVs from unconditioned THP-1 macrophages (MAC EVs) for 24 and 48 h is shown. Data correspond to MAC EVs derived from **(C)** one million and **(D)** two million cells and did not show significant changes in proliferation relative to the control. Cells cultured in complete mediums without EVs served as the control group. Statistical significance was determined using two-way ANOVA with Dunnett’s post-test, where *p < 0.05 and **p < 0.01. Data are presented as mean ± SEM of the percentage proliferation relative to control from three independent experiments performed in triplicate.

To explore the impact of TNF-α-conditioned macrophage-derived extracellular vesicles (TNF EVs) on the growth of ER+ breast cancer cells, we evaluated the proliferation of MCF-7 cells after exposure to different TNF EV subpopulations and concentrations. We first analyzed the uptake of TNF EVs by MCF-7 cells and observed that these cells efficiently uptake the vesicles (Supplementary Figure S1). After confirming the successful uptake of EVs, MCF-7 cells were seeded in 96-well plates with an equal number of cells in each well and grown in a complete medium at 37°C. After 24 h of initial culture, we introduced TNF EVs derived from 2 K, 10 K, 100 K, and 200 K ultracentrifugation fractions at two different concentrations (EVs isolated from 1 or 2 × 10^6^ cells). We included EVs from unconditioned macrophages (MAC EVs) for control purposes to determine whether the observed effects were specific to TNF EVs (Figure 2). Cell proliferation was evaluated using the Alamar Blue assay. Measurements were taken 24 and 48 h after EV treatment to assess temporal effects on cell proliferation. Our results indicated a significant, dose-dependent increase in MCF-7 cells proliferation after 24 h treatment with TNF EVs coming from 2 × 10^6^ cells, particularly with the 2 K, 100 K, and 200 K subpopulations. Interestingly, the enhanced proliferation observed at 24 h was not sustained at 48 h. We hypothesize that the proliferative effect of TNF EVs might require a continuous supply of EVs to maintain this stimulation. This hypothesis is supported by the fact that after 24 h, the medium was washed and replaced with fresh medium containing Alamar Blue reagent, potentially limiting further EV-mediated stimulation. In contrast, MCF-7 cells treated with MAC EVs exhibited no significant changes in proliferation at any time point or concentration, reinforcing the specific role of TNF conditioning in driving the proliferative response.

These results suggest that TNF EVs, especially the 2 K, 100 K, and 200 K subpopulations, promote dose-dependent proliferation of ER+ breast cancer cells and may require continuous exposure to sustain this effect. This highlights the role of macrophage-derived EVs in tumor growth and points to EV-mediated signaling as a potential therapeutic target to limit breast cancer proliferation.

### 3.3 TNF EVs promote migration of breast tumor cells

**FIGURE 3:**
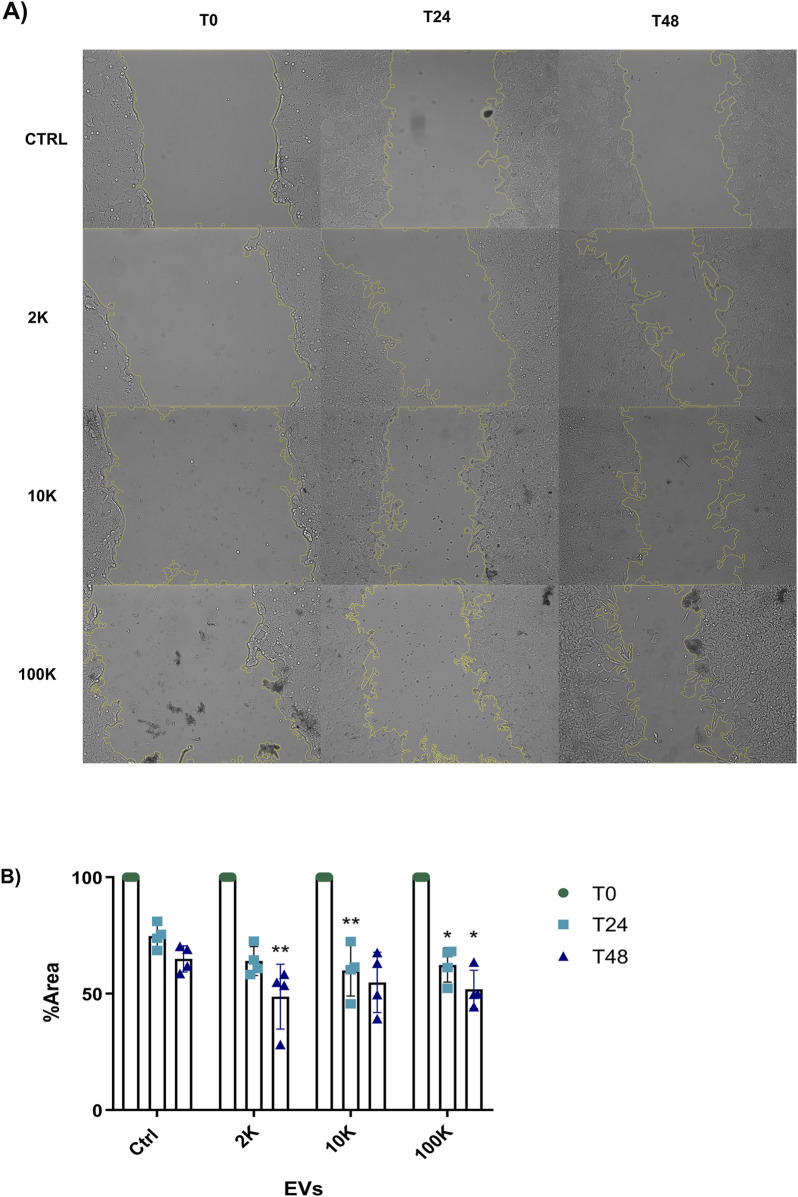
MCF-7 migration is promoted by TNF EVs **(A)** Representative images from the wound healing assay show MCF-7 cell migration at 24 and 48 h following treatment with EVs derived from TNF-α conditioned THP-1 macrophages. EV subpopulations (2 K, 10 K, and 100 K) were added at a concentration of 16,000 vesicles per target cell. **(B)** Quantitative analysis of wound closure was measured at three positions in duplicate for each condition and compared to control, where no EVs were added. Wound closure is expressed as a percentage of the remaining area relative to the control, with 100% representing the initial wound area at time zero. Statistical significance was determined by two-way ANOVA followed by Dunnett’s post-test *p < 0.05, **p < 0.01. Data are presented as mean ± SD from two independent experiments conducted in duplicate.

Following our observations that TNF EVs enhance the proliferation of MCF-7 ER+ breast cancer cells, we next assessed their impact on cell migration. To do this, we performed a wound-healing assay to determine whether TNF EVs could promote the migratory behavior of MCF-7 cells, a key step in cancer metastasis. MCF-7 cells were cultured in 24-well plates, and wounds were created in confluent monolayers. TNF EVs were added at a rate corresponding to 16,000 vesicles per target cell. The medium was supplemented with 1% FBS to limit proliferation and ensure that any wound closure observed was predominantly due to migration rather than cell division. Imaging of wound closure was performed at 24 and 48 h. Our data demonstrated that TNF EVs significantly promoted wound closure in MCF-7 cells compared to control conditions, where no EVs were added. All TNF EVs subpopulations tested (2 K, 10 K, 100 K) enhanced migration of MCF-7 cells, leading to faster wound closure. This effect was more pronounced at 48 h, with a clear increase in cell migration observed in the presence of TNF EVs relative to untreated controls (Figure 3). Notably, the migration-promoting effect of TNF EVs was maintained throughout the 48-h assay, as EVs were continuously present. These results suggest that TNF EVs could actively contribute to the enhanced migratory potential of breast cancer cells, supporting their role in facilitating metastatic progression.

### 3.4 TNF EVs induce morphological changes in MCF-7 cells

**FIGURE 4:**
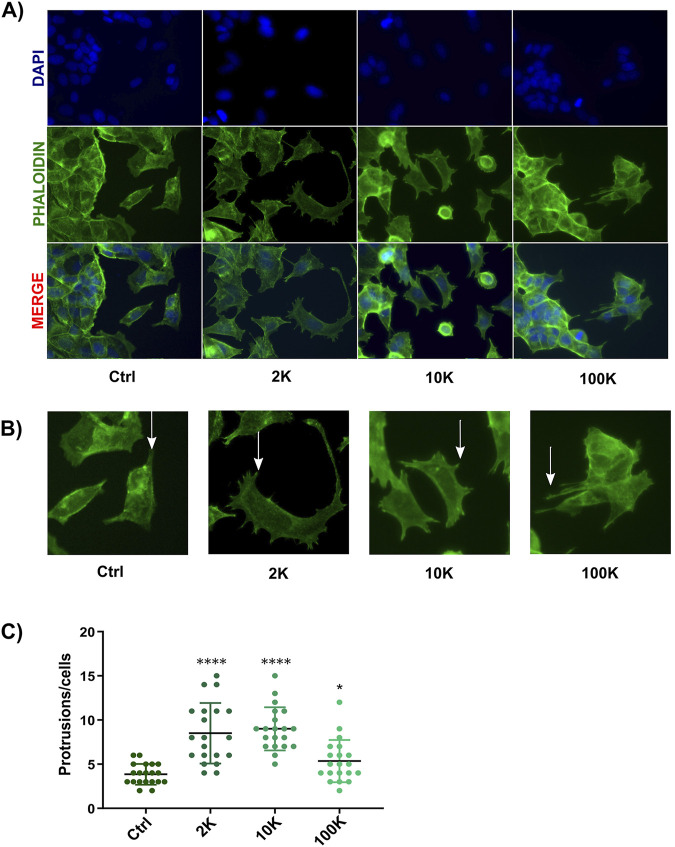
Analysis of morphological changes in MCF-7 cells. **(A)** After treatment with TNF EVs, confocal microscopy images of MCF-7 cells showed actin filaments stained with Phalloidin (green) and nuclei stained with DAPI (blue). The bottom row presents the overlay of both stainings. **(B)** Representative images indicating cellular protrusions, marked by arrows, in MCF-7 cells treated with TNF EVs compared to control cells. **(C)** The protrusions were quantified using ImageJ software, with protrusions counted in 20 cells per condition. Statistical significance was determined by one-way ANOVA with Dunnett’s post-test *p < 0.05; ****p < 0.0001 compared to the control condition. Data are presented as the mean ± SD of protrusions per cell.

In addition to their role in promoting migration, TNF EVs were found to induce morphological changes in MCF-7 cells. This observation was made during the wound healing assay, where MCF-7 cells treated with TNF EVs exhibited a significant increase in cellular protrusions compared to untreated control cells. These morphological changes are closely related to a more migratory and invasive phenotype, which is characteristic of epithelial-mesenchymal transition (EMT) and indicates enhanced metastatic potential in cancer cells. To quantify these changes, MCF-7 cells were fixed after performing the wound healing assay and stained with Phalloidin 568, a fluorescently labeled molecule that binds specifically to filamentous actin (F-actin), providing a clear view of the cytoskeletal structure. Furthermore, DAPI was used to stain the nuclei for easier identification and counting of cells. Confocal microscopy images revealed that cells treated with TNF EVs displayed a marked increase in the number of cellular protrusions, consistent with the formation of actin driven structures that promote motility (Nurmagambetova et al., 2023). In contrast, control cells without TNF EVs treatment maintained a more typical epithelial morphology with fewer protrusions, indicative of a less motile, more adherent profile. This observation suggests that TNF EVs play a significant role in remodeling the cytoskeleton of MCF-7 cells, likely through the activation of pathways involved in cell motility. This also indicates that TNF EVs promote not only migration but also structural changes that support the transition of cancer cells to a more aggressive phenotype (Figure 4).

### 3.5 TNF EVs induce alterations in EMT markers in MCF-7 cells

**FIGURE 5:**
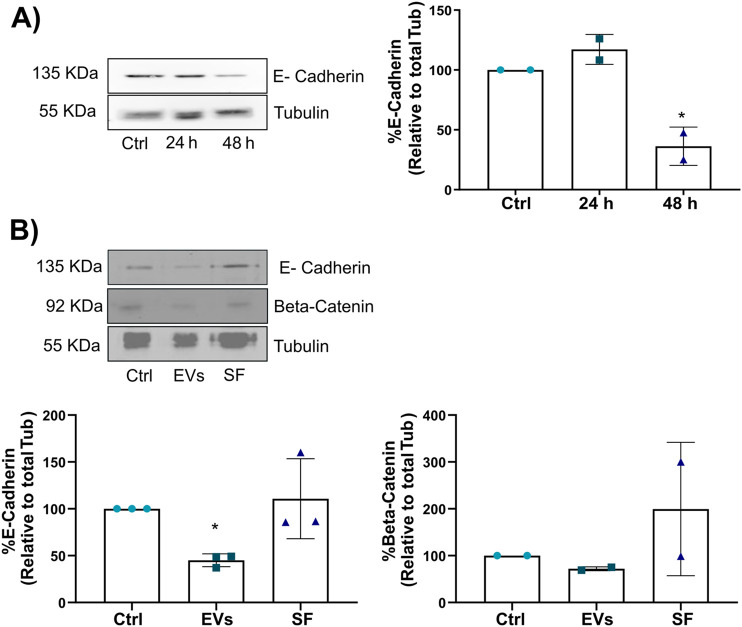
TNF EVs promote EMT in MCF-7 cells. **(A)** Western blot of MCF-7 cell lysates following treatment with TNF EVs for 24 and 48 h, showing a significant reduction in E-cadherin expression at 48 h. Tubulin was used as a loading control. **(B)** Western blot of MCF-7 cell lysate after 48 h of treatment with TNF EVs or SF, with no observable changes in E-cadherin or β-catenin expression in the SF fraction, indicating that EVs exclusively trigger the effects. Tubulin was used as a loading control. Densitometric analysis of the Western blot bands shows the relative protein levels of E-cadherin and β-catenin, normalized to Tubulin. Statistical significance was calculated using one-way ANOVA with Dunnett’s post-test *p < 0.05. Data represent the mean ± SD from two or three independent experiments.

Given that TNF EVs promote MCF-7 breast cancer cell migration, we investigated whether these vesicles could also induce EMT, a process critical for tumor progression and metastasis. EMT is characterized by the downregulation of epithelial markers, such as E-cadherin, and the upregulation of mesenchymal features, which facilitate enhanced motility and invasiveness in cancer cells (Drasin et al., 2011; Wang and Zhou, 2011). MCF-7 cells were treated with TNF EVs for 24 and 48 h, followed by Western blot analysis of key EMT markers. At 24 h, no significant changes were detected in the expression of E-cadherin, which is critical for maintaining epithelial cell-cell adhesion (Figure 5A). However, after 48 h of TNF EV exposure, a significant reduction in the levels of both E-cadherin and β-catenin was observed, indicating that TNF EVs trigger an EMT process in MCF-7 cells. Additionally, no significant alterations in E-cadherin or β-catenin levels were observed in the cells treated with the soluble fraction (SF). Controlling the enriched protein fraction confirms that TNF EVs directly modulate intracellular EMT signaling pathways. These findings, in addition to the above described, suggest that TNF EVs play a crucial role in promoting EMT, thereby enhancing the invasive potential of breast cancer cells (Figure 5B).

### 3.6 TNF EVs induce a transition towards a tumor stem cell-like phenotype in MCF-7 cells

**FIGURE 6:**
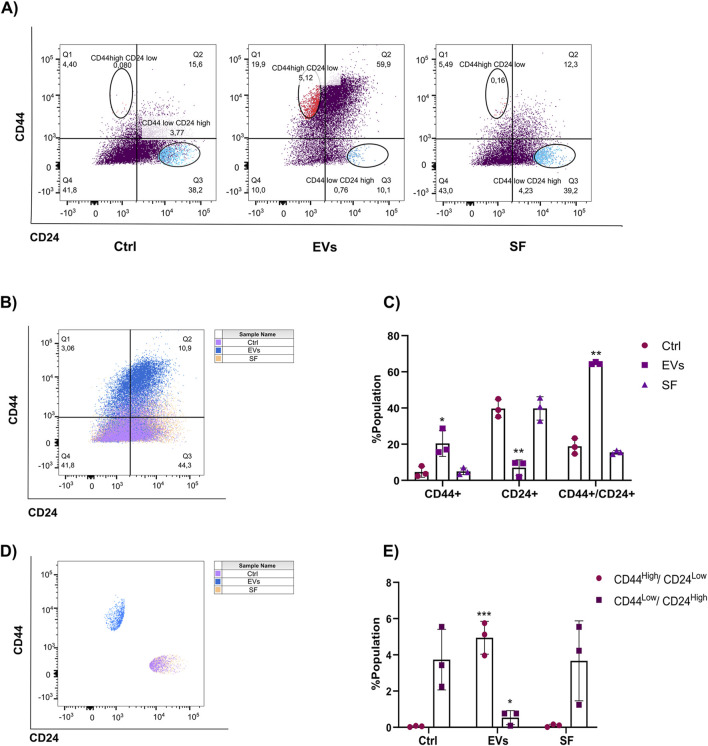
TNF EVs promote the expression of stem cell markers in MCF-7 cells. **(A)** Flow cytometry analysis of MCF-7 cells incubated with TNF EVs for 48 h and then stained with CD44-APC and CD24-PECy7 antibodies. Untreated cells and cells treated with the SF were used as controls. **(B)** Representative dot plots show CD44 and CD24 expression distribution in different treatment groups. **(C)** Quantification of the percentage of the population expressing different markers presented in **(B)**. **(D)** Representative dot plots showing that TNF EV-treated cells demonstrated a clear shift toward the CD44^High^/CD24^Low^ phenotype, while control and SF-treated cells maintained a typical CD44^Low^/CD24^High^ expression profile. **(E)** Quantifying the percentage of the population expressing CD44^High^/CD24^Low^ and CD44^Low^/CD24^High^. Statistical significance was determined using two-way ANOVA with Dunnett’s post-test *p < 0.05; **p < 0.01; ***p < 0.001. Data represent mean ± SD from a representative experiment performed in triplicate.

Tumor stem cells are known for their role in cancer initiation, metastasis, and therapeutic resistance. These cells, characterized by their ability to self-renew and differentiate, are often associated with poor prognosis in breast cancer due to their enhanced metastatic potential and capacity to evade conventional therapies (Mani et al., 2008; Shan et al., 2021; Sheridan et al., 2006). Given that TNF EVs promote EMT in MCF-7 cells, we hypothesized that TNF EVs might also drive the acquisition of a tumor stem cell-like phenotype. This phenotype is commonly associated with a CD44^High^/CD24^Low^ expression profile, which has been linked to increased tumorigenicity, resistance to endocrine therapies, and disease recurrence in breast cancer. To investigate this, we treated MCF-7 cells with TNF EVs for 48 h and subsequently analyzed the expression of stem cell surface markers CD44 and CD24 using flow cytometry. Cells were stained with anti-CD44-APC and anti-CD24-PECy7 antibodies to assess the proportion of cells exhibiting the CD44^High^/CD24^Low^ phenotype. As controls, cells were treated with the SF, and untreated cells were included for baseline comparisons. Our flow cytometry analysis revealed a significant increase in the population of cells expressing CD44, a decrease in CD24, and an increase in CD44+/CD24+ cells in MCF-7 cells treated with TNF EVs compared to control and SF-treated cells (Figures 6A–C). Additionally, we observed a significant increase in the population expressing the CD44^High^/CD24^Low^ phenotype in the TNF EV-treated group compared to both the untreated control and SF-treated cells (Figures 6D,E). This shift in cell surface marker expression suggests that TNF EVs actively promote a transition toward a tumor stem cell-like state, highlighting their role as key modulators of the tumor microenvironment. Interestingly, the soluble fraction did not induce similar changes, confirming that the observed effects are specifically mediated by TNF EVs rather than by soluble factors released during the isolation process. To investigate the functional consequences of this shift toward a stem-like phenotype, we employed a 3D tumor spheroid formation assay using ultra-low attachment conditions. MCF-7 cells were cultured as spheroids in the presence or absence of TNF EVs, and spheroid formation was assessed after 6 days. TNF EV-treated cells formed a significantly higher number of spheroids compared to untreated controls (Supplementary Figures S2A,B), suggesting an enhanced capacity for independent growth—a characteristic of stem-like cells (Chen et al., 2022; Erden Tayhan, 2024; Nayak et al., 2023; Nath and Devi, 2016). Upon completion of the culture period, spheroids were collected, enzymatically dissociated into single-cell suspensions, and analyzed by flow cytometry for CD44 and CD24 expression. Consistent with our 2D findings, spheroid-derived cells from the TNF EV-treated group displayed an increase in the CD44^High^/CD24^Low^ subpopulation, along with a rise in CD44+/CD24+ double-positive cells (Supplementary Figures S2C). These results indicate that TNF EVs also promote features associated with tumor stemness in 3D culture, further supporting their role as modulators of cell plasticity within the breast cancer microenvironment.

### 3.7 TNF EVs and their role in endocrine resistance in MCF-7 breast cancer cells

**FIGURE 7:**
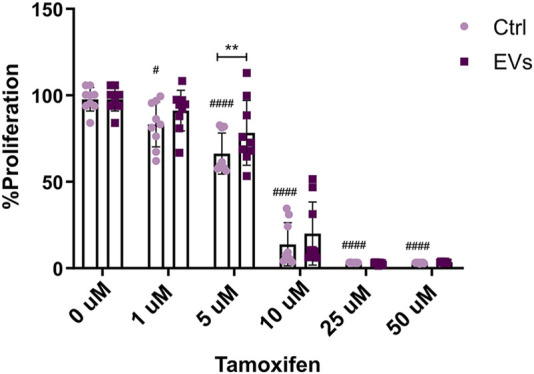
TNF EVs impair the MCF-7 sensibility to Tamoxifen. MCF-7 cells were treated with TNF EVs for 24 h, followed by the addition of increasing concentrations of Tamoxifen (0, 1, 5, 10, 25, and 50 μM). Cell proliferation was evaluated using the Alamar Blue assay. Bar graph representing the percentage of MCF-7 cell proliferation in the presence of TNF EVs compared to control cells treated with Tamoxifen alone. Statistical analysis was performed using two-way ANOVA followed by Dunnett’s post-test *p < 0.05, **p < 0.01 ^#^p < 0.05, ^####^p < 0.0001. Data represent the mean ± SD from three independent experiments performed in triplicate.

Endocrine resistance, whether *de novo* or acquired, remains a major obstacle in the treatment of ER+ breast cancer. Resistance to Tamoxifen poses a significant clinical challenge, allowing tumor cells to proliferate even in the presence of the drug. Given the observed effects of TNF EVs promoting migration and inducing a tumor stem cell-like phenotype in MCF-7 cells, we sought to explore whether these vesicles also contribute to endocrine resistance. To test this, we treated MCF-7 cells with TNF EVs and exposed them to different concentrations of Tamoxifen (0, 1, 5, 10, 25, and 50 μM). Experiments were conducted in a low-serum (1% FBS) and phenol red-free medium to minimize estrogen receptor activation by serum components. Proliferation was assessed after 24 h using Alamar Blue assay as described previously. Our results suggest that the presence of TNF EVs significantly enhanced the proliferation of MCF-7 cells treated with Tamoxifen, particularly at the 5 μM concentration, compared to control cells treated only with Tamoxifen. These observations indicate that TNF-EVs may counteract the inhibitory effects of Tamoxifen, thereby enabling cancer cells to persist in a drug-resistant state and continue their proliferation. Notably, cells treated with higher concentrations of Tamoxifen (25 μM and 50 μM) still showed signs of reduced proliferation, although the protective effect of TNF EVs was evident at lower concentrations (Figure 7).

These findings suggest that TNF EVs are involved in the mechanisms of endocrine resistance in breast cancer cells, potentially through their ability to alter cellular responses to Tamoxifen.

### 3.8 Role of MCF-7 EVs in macrophage polarization

**FIGURE 8:**
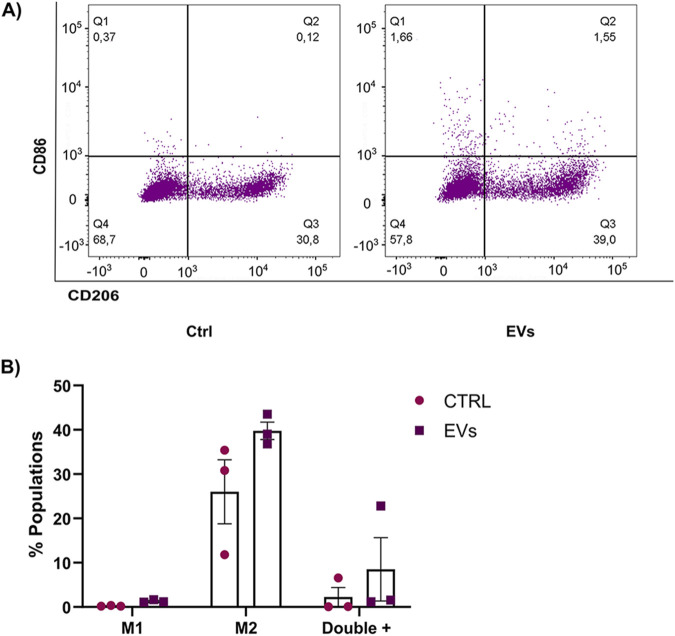
MCF-7 derived EVs modulate macrophage profile. **(A)** Flow cytometry analysis of THP-1 macrophages incubated with MCF-7 EVs for 48 h, stained with CD86-APC (M1 marker) and CD206-APCCy7 (M2 marker). Untreated cells were used as controls. **(B)** Quantifying the percentage of macrophages expressing CD86 and CD206 after treatment with MCF-7 EVs compared to untreated controls. Data are presented as the mean ± SD from three independent experiments.

Macrophages are a key component of the tumor microenvironment and play a dual role in tumor progression. Depending on their polarization phenotype, macrophages can either exhibit pro-inflammatory, anti-tumor functions (M1 phenotype) or adopt an immunosuppressive, pro-tumoral role (M2 phenotype). Tumor-derived EVs have been implicated in reprogramming macrophages toward the M2 phenotype, which promotes tumor growth, immune evasion, and metastasis. In this study, we sought to determine whether EVs derived from MCF-7 breast cancer cells (MCF-7 EVs) could modulate macrophage polarization. THP-1 monocytes were differentiated into macrophages using PMA and subsequently treated with MCF-7 EVs for 48 h. To assess macrophage polarization, we measured the expression of CD86 (a surface marker of M1 macrophages) and CD206 (a surface marker of M2 macrophages) by flow cytometry. Non-activated macrophages M0 were used as controls to evaluate the baseline expression of these markers. Our results show that treatment with MCF-7 EVs resulted in a slight increase in the population of macrophages expressing both M1 and M2 markers, suggesting that MCF-7 EVs may induce a mixed polarization state. We noticed an increase in the CD206-positive (M2-like) macrophage population. This type of macrophage is often linked to functions that promote tumors, such as increasing blood vessel growth, suppressing the immune system, and aiding metastasis (Figure 8). Despite observing an increase in M2 polarization, the results were not statistically significant compared to the controls. This suggests that the impact of MCF-7 EVs on macrophage polarization may be nuanced or necessitate extended exposure or higher concentrations of EVs to be fully evident. These findings underscore the intricate nature of macrophage plasticity and indicate a need for further investigations into the mechanisms by which tumor-derived extracellular vesicles influence immune responses within the tumor microenvironment.

### 3.9 MCF-7 EVs increase expression of PD-1 in macrophages

**FIGURE 9:**
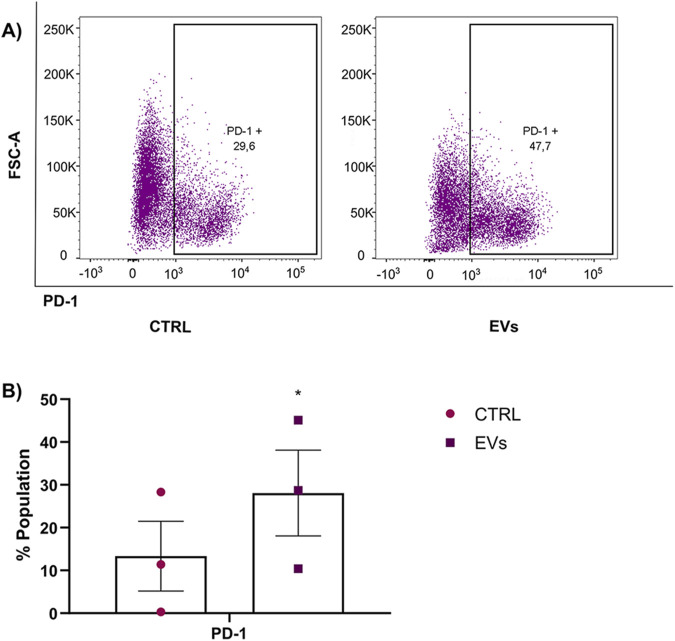
PD-1 expression in macrophages was enhanced by treatment with MCF-7 EVs. **(A)** THP-1 macrophages were incubated with MCF-7-derived EVs for 48 h and stained with PD-1-PECy7 antibodies. PD-1 expression was measured using flow cytometry, and untreated macrophages served as the control group. **(B)** Quantification of the percentage of macrophages expressing PD-1 in EV-treated versus control groups. Data are presented as mean ± SD from three independent experiments. Statistical significance was determined using a t-test *p < 0.05.

Cancer immunotherapy is a novel and promising approach. Still, unfortunately, one of the reasons this therapy may also fail is due to the inhibition of immune checkpoints (Hanahan and Weinberg, 2011) such as programmed death ligand 1/2 (PD-L1/2) and programmed death receptor 1 (PD-1) or CD80, which are secreted by macrophages. PD-1 is a recognized immune checkpoint receptor that, upon expression on macrophages, correlates with immunosuppressive functions and suboptimal anti-tumor immune responses (Li et al., 2023; Arlauckas et al., 2017; Chen et al., 2013; Gordon et al., 2017). Elevated PD-1 levels in TAMs have been correlated with reduced phagocytosis, impaired activation, and enhanced tumor progression (Gordon et al., 2017; Kono et al., 2020; Xu et al., 2023; Zhang et al., 2023) Given this background, we investigated whether MCF-7 EVs could induce PD-1 expression in macrophages, potentially contributing to immune evasion. THP-1 monocytes were differentiated into macrophages and subsequently treated with MCF-7 EVs for 48 h to explore this. Flow cytometry was performed to measure the expression of PD-1 on macrophages after EVs treatment. Untreated macrophages served as the control to establish baseline PD-1 expression. Our results demonstrated that treatment with MCF-7 EVs significantly increased the expression of PD-1 on macrophages compared to untreated control cells (Figure 9). The ability of MCF-7 EVs to induce immune checkpoint receptor expression in macrophages aligns with previous findings that tumor-derived EVs can reprogram immune cells, pushing them toward a phenotype that supports tumor growth and metastasis (Kugeratski and Kalluri, 2020). The increased PD-1 expression in EV-treated macrophages could also have broader implications for breast cancer therapy.

## 4 Discussion

Breast cancer remains a significant challenge in oncology, being one of the most common malignancies and a leading cause of morbidity and mortality among women worldwide. With an increasing incidence, it is clear that efforts in both scientific research and clinical practice are essential to address this global health burden. The complexity of breast cancer lies not only in the heterogeneity of the disease itself but also in the dynamic interactions within the tumor microenvironment (TME). The TME comprises a diverse range of components, including immune cells, stromal cells, soluble factors, and EVs, all of which play crucial roles in shaping tumor behavior, metastasis, and response to therapies. Approximately 70% of breast tumors are classified as estrogen receptor-positive (ER+), making ER critical therapeutic targets in breast cancer treatment. Therapies such as selective estrogen receptor modulators (e.g., Tamoxifen) and aromatase inhibitors have significantly improved survival rates in ER+ breast cancer patients. However, one of the major challenges in treating ER+ breast cancer is the development of resistance to endocrine therapies, either *de novo* or acquired during treatment. This resistance remains a critical issue in clinical oncology, driving relapse and metastasis in a significant proportion of patients.

Our study sought to understand the underlying mechanisms contributing to endocrine resistance, focusing on the role of TNF-α conditioned macrophage-derived EVs in this process. Specifically, we investigated the effects of TNF EVs on breast cancer cells’ proliferation, migration, and therapy resistance. Previous work from our group demonstrated that macrophages when exposed to a pro-inflammatory environment such as TNF-α, adopt a pro-tumoral phenotype that supports tumor progression (Castellaro et al., 2019). Our current research expands on this concept by investigating the role of macrophage-derived extracellular vesicles (EVs) in modulating endocrine resistance.

The TME plays a pivotal role in regulating tumor behavior and the response to different therapies. Within this complex milieu, EVs have emerged as key mediators of cellular communication, capable of transporting bioactive molecules such as proteins, lipids, and nucleic acids. These vesicles not only facilitate crosstalk between tumor cells but also modulate the behavior of stromal and immune cells within the TME. Given their capacity to influence processes such as cell proliferation, migration, and immune responses, EVs are increasingly recognized as critical regulators of tumor progression and therapy resistance (Demory et al., 2013; Koga et al., 2005; O’Brien et al., 2013; Skog et al., 2008).

Our study optimized two reproducible protocols for EVs isolation, ensuring that the obtained vesicles were enriched and characterized according to established guidelines (Welsh et al., 2024; Witwer et al., 2021). However, it is important to note that even with optimized protocols, the potential for contamination with other particles or proteins cannot be entirely excluded (Mathieu et al., 2019). Nevertheless, the fractions of EVs we acquired were highly enriched and thoroughly characterized, confirming their identity and enabling us to continue with functional analyses.

One of the key findings of our study is the ability of TNF EVs to enhance the proliferative and migratory capacities of ER+ breast cancer cells. The wound healing assay demonstrated that TNF EVs significantly promote the migration of breast cancer MCF-7 cells. The morphological changes we observed in EV-treated breast cancer MCF-7 cells, such as the formation of cellular protrusions, further support the idea that EVs contribute to a more migratory phenotype. These findings are particularly important in the context of metastasis, as the ability of cancer cells to migrate and invade surrounding tissues is a hallmark of metastatic disease. Our findings indicate that TNF-derived extracellular vesicles play a pivotal role in facilitating epithelial-to-mesenchymal transition. During this process, epithelial cells undergo a loss of adhesion properties and acquire mesenchymal characteristics, which significantly enhances their migratory and invasive capabilities. EMT is a key event in cancer metastasis, and the observation that TNF EVs can induce EMT-associated morphological changes in MCF-7 cells highlights the potential role of EVs in driving metastasis in breast cancer.

In addition to morphological changes, we explored the molecular mechanisms underlying the EV-induced migratory phenotype by assessing the expression of EMT markers. Our data reveal that TNF EVs induce the downregulation of epithelial markers such as E-cadherin and β-catenin. These changes indicate EMT, further supporting the notion that EVs play a role in promoting metastasis. It is important to note that the observed effects were attributed to the enriched fraction of EVs, not to other soluble components present in the conditioned media. EVs’ ability to modulate EMT marker expression has been previously reported in various cancer models (Huang et al., 2022; Syn et al., 2016). Our research introduces a novel aspect to this domain by illustrating that extracellular vesicles derived from macrophages exposed to a pro-inflammatory milieu can effectively trigger epithelial-mesenchymal transition in breast cancer cells. This finding underscores the importance of the tumor microenvironment in shaping cancer cell behavior, suggesting that targeting EV-mediated signaling pathways could be a potential therapeutic strategy to prevent metastasis.

Our study also sheds light on the role of TNF EVs in expanding the population of tumor stem cells within the breast cancer cell line MCF-7. Tumor stem cells, characterized by their CD44^High^/CD24^Low^ expression profile, are known to drive tumor progression, metastasis, and therapy resistance (Sheridan et al., 2006; Ghuwalewala et al., 2016; Poothakulath Krishnan et al., 2023). The expansion of this stem cell-like population in EV-treated cells suggests that EVs play a direct role in maintaining the tumor stem cell population, contributing to the aggressive phenotype of breast cancer. In line with this, EV-treated cells demonstrated a greater capacity to form mammospheres, a functional test that reflects self-renewal abilities and the presence of stem-like cells. Notably, tumor stem cells frequently show resistance to standard therapies, including endocrine treatments like Tamoxifen (Rodriguez et al., 2019; O’Brien et al., 2011).

Endocrine resistance is a multifaceted problem, with multiple mechanisms contributing to the failure of therapies such as Tamoxifen. These mechanisms include alterations in ER signaling, activation of alternative survival pathways (e.g., PI3K/AKT), and changes in the tumor microenvironment (Rani et al., 2019). Our study provides evidence that EVs released by TNF-conditioned macrophages are involved in this process, promoting sustained proliferation even in the presence of Tamoxifen. This observation is particularly relevant, given that endocrine resistance remains a major challenge in the treatment of ER+ breast cancer. Previous studies have linked EVs to the process of drug resistance by transferring miRNAs and proteins that modulate drug response (Chen et al., 2014; Xu et al., 2016; La Camera et al., 2021; Semina et al., 2018; Liu et al., 2021). Our research strongly supports this evidence, indicating that focusing on EV-mediated communication could provide an innovative strategy to address therapy resistance in breast cancer.

In addition to their role in tumor progression and drug resistance, EVs are emerging as key players in modulating the immune response within the TME. Tumor-associated macrophages (TAMs), often polarized toward an immunosuppressive M2 phenotype, contribute to immune evasion and tumor growth. Our study investigated whether EVs from MCF-7 cells could modulate macrophage polarization. We found that EV-treated macrophages displayed an increase in both M1 and M2 markers, suggesting a mixed polarization state. More importantly, we observed a significant increase in PD-1 expression on EV-treated macrophages. PD-1 is an immune checkpoint receptor that inhibits macrophage activation and phagocytosis, promoting immune suppression within the TME. The upregulation of PD-1 in macrophages suggests that EVs may contribute to creating an immunosuppressive environment, a key feature of breast cancer progression (Gordon et al., 2017; Kono et al., 2020; Xu et al., 2023; Lu et al., 2019).

Checkpoint inhibitors targeting PD-1/PD-L1 have shown promise in restoring immune activity against tumors, but the presence of PD-1-expressing macrophages within the TME may reduce the efficacy of these therapies. Our findings suggest that tumor-derived EVs could hinder the success of immunotherapies by promoting the immunosuppressive functions of TAMs. By combining therapies specifically aimed at enhancing the effects of extracellular vesicles with immune checkpoint inhibitors, we may be able to offer breast cancer patients a more effective treatment option. While our study provides valuable insights into the role of EVs in breast cancer progression and resistance, future research should address several limitations. The molecular mechanisms by which EVs exert their effects on cancer and immune cells remain only partially understood. Further studies are needed to identify the specific cargo carried by TNF EVs, such as miRNAs, proteins, or lipids, and to determine how these molecules mediate the observed effects. Although our experiments were conducted *in vitro*, it is essential to validate these findings *in vivo* using animal models of breast cancer. *In vivo,* studies would provide a more comprehensive understanding of how EVs influence tumor behavior and immune responses within the context of the entire organism. Additionally, studying the biodistribution of EVs in animal models would clarify how these EVs interact with different tissues and contribute to metastasis. While our study focused on the effects of EVs on breast cancer cells and macrophages, EVs likely influence other cell types within the TME, such as fibroblasts, endothelial cells, and lymphocytes. Investigating the broader impact of EVs on the tumor microenvironment will provide a more complete picture of how these vesicles contribute to tumor progression. Additionally, early detection remains crucial for improving treatment outcomes and disease progression. In this regard, EVs are emerging as promising biomarkers due to their stability in biological fluids and capacity to reflect the molecular characteristics of their cells of origin (Bandu et al., 2024; Dorado et al., 2024; Ochiya et al., 2024). This opens exciting new avenues for early diagnosis and the development of novel therapeutic approaches that aim to modulate intercellular communication within the TME, with the ultimate goal of improving clinical outcomes for breast cancer patients.

## 5 Conclusion

In summary, our study emphasizes the vital role of EVs derived from TNF-α conditioned macrophages in advancing breast cancer progression, metastasis, and endocrine resistance. These EVs are essential mediators of intercellular communication within the tumor microenvironment, enabling the transfer of oncogenic signals and traits linked to drug resistance. Their capacity to trigger EMT, increase the tumor stem cell population, and alter immune responses highlights their significance in influencing tumor behavior. Given their contribution to therapy resistance and immune evasion, targeting EV-mediated communication offers a promising therapeutic approach for breast cancer. Disrupting oncogenic signaling networks that promote tumor growth and metastasis could be achieved by inhibiting EV release or blocking their uptake by recipient cells. Furthermore, combining EV-targeted therapies with current treatments, such as immune checkpoint inhibitors, may improve clinical outcomes and diminish the risk of therapy resistance. As research into EVs advances, it becomes increasingly clear that these vesicles are not just products of cellular activities but dynamic players in tumor biology. Their potential as both biomarkers for early detection and therapeutic targets opens up exciting new avenues for enhancing breast cancer treatment and improving patient outcomes.

## Data Availability

The datasets presented in this study can be found in online repositories. The names of the repository/repositories and accession number(s) can be found in the article/Supplementary Material.

## Glossary

AIs: Aromatase Inhibitors
ATCC: American Type Culture Collection
DMEM: Dulbecco’s modified Eagle Medium
DNA: deoxyribonucleic acid
DTT: Dithiothreitol
E2: Estradiol
ECM: Extracellular Matrix
EGF: Epidermal Growth Factor
EMT: Epithelial- Mesenchymal Transition
ER: Estrogen Receptor
EREs: Estrogen Response Elements
ERK: Extracellular-Signal-Regulated Kinase
EVs: Extracellular Vesicles
FBS: Fetal Bovine Serum
FGF: Fibroblast Growth Factor
FSC-A: Forward Scatter Area
IFN-γ: Interferon Gamma
IL: Interleukin
LPS: Lipopolysaccharide
M1: Macrophages polarized towards an M1 profile
M2: Macrophages polarized towards an M2 profile
MVBs: Multivesicular Bodies. Cuerpos Multivesiculares
NF-κB: Nuclear Factor kappa B
NTA: Nanoparticle tracking analysis
PBS: Phosphate buffered saline
PD-1: Programmed cell death protein 1
PD L1: Programmed cell death ligand protein 1
PFA: Paraformaldehyde
PMA: Phorbol 12-myristate 13-acetate
PSN: Penicillin-streptomycin-neomycin
RT: Room Temperature
SEC: Size Exclusion Chromatography
SF: Soluble fraction
SERDs: Selective Estrogen Receptor Degrader
SERMs: Selective Estrogen Receptor Modulators
TAMs: Tumor‐ associated macrophages
TEM: Transmission Electron Microscopy
TME: Tumor Microenvironment
TNF-α: Tumoral Necrosis Factor alpha.
