# Supplementary figures and images for: Intercellular communication between extracellular vesicles from conditioned macrophages and breast cancer cells drives endocrine therapy resistance
Source: Front Cell Dev Biol. 2025 Jun 3;13:1548724. doi: 10.3389/fcell.2025.1548724 (PMC12188363; doi:10.3389/fcell.2025.1548724)

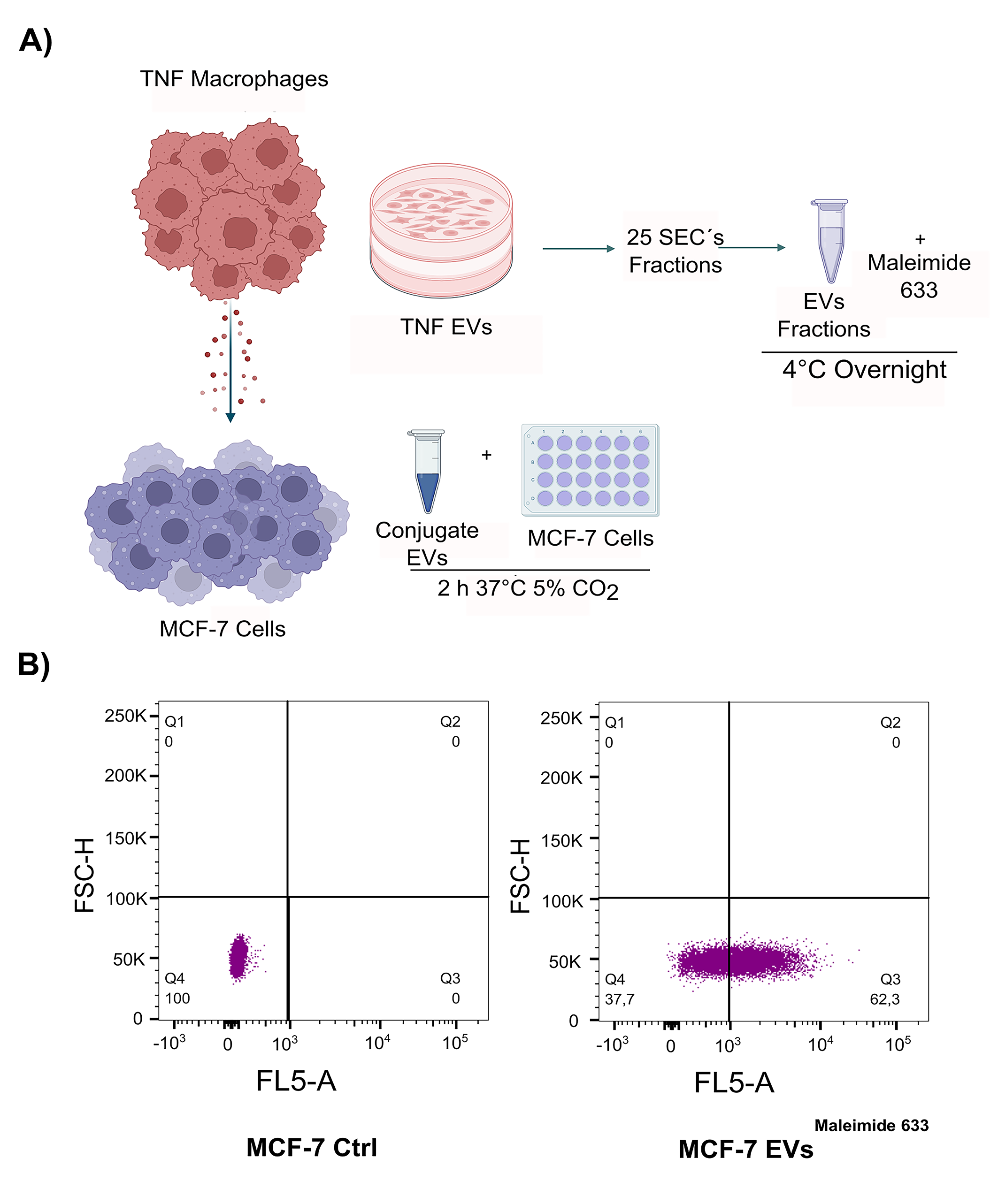

Supplement: Supplementary file 1 [file Image1.tiff]

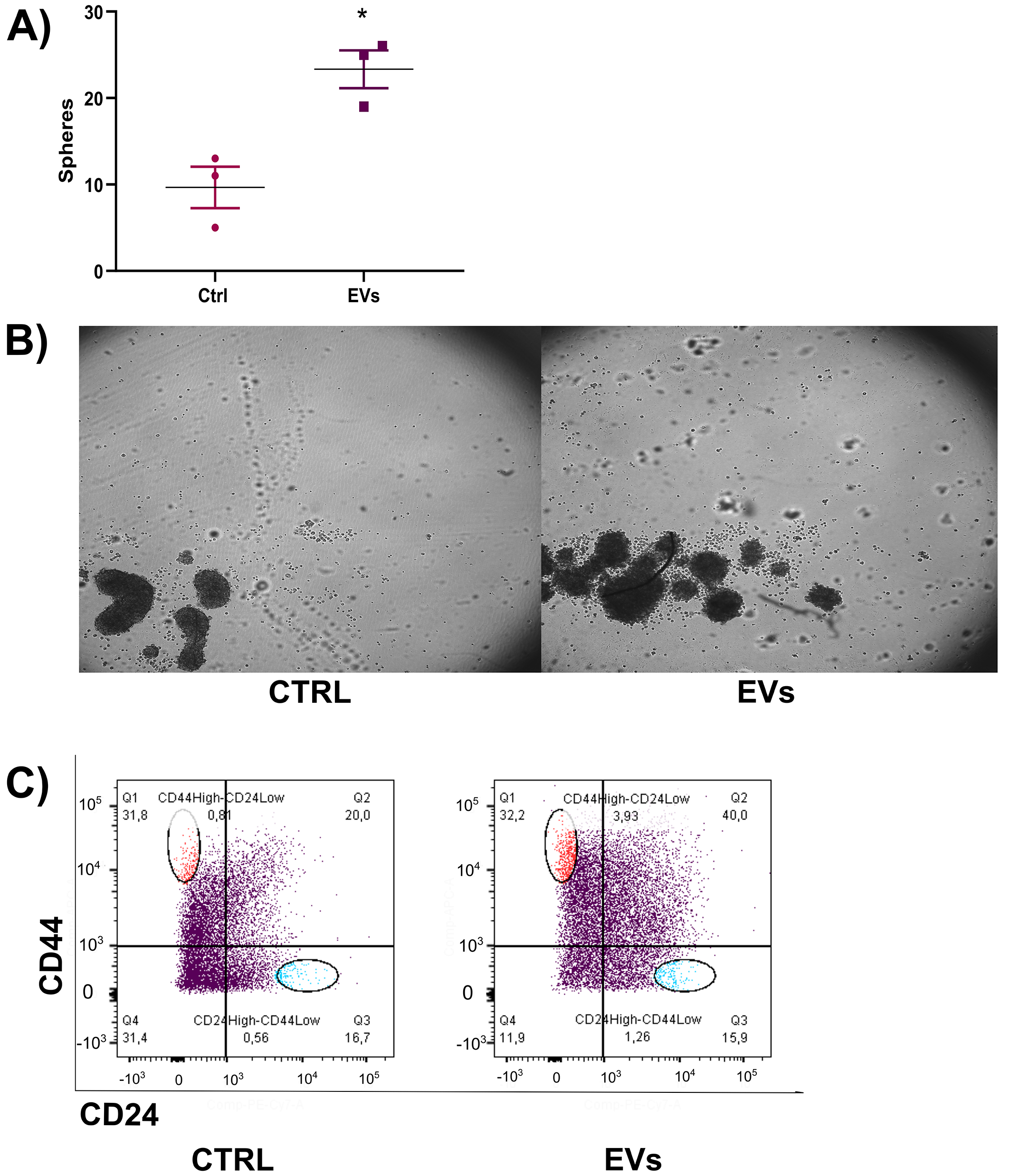

Supplement: Supplementary file 2 [file Image2.tif]
